# A Case of Persistent Diarrhea and Fevers Uncovering Colorectal Adenocarcinoma

**DOI:** 10.7759/cureus.101745

**Published:** 2026-01-17

**Authors:** Amber J Stout, Michael Medina, Brian Rios, Nayle Araguez-Ancares

**Affiliations:** 1 School of Medicine, St. George's University School of Medicine, St. George's, GRD; 2 School of Medicine, Ross University School of Medicine, Bridgetown, BRB; 3 School of Medicine, American University of the Caribbean, Cupecoy, SXM; 4 Department of Internal Medicine, Jackson Memorial Hospital, Miami, USA

**Keywords:** atypical cancer presentation, colon cancer surveillance, colonoscopy quality indicators, colorectal cancer, non-bloody diarrhea, risk factors for colorectal cancer

## Abstract

Colorectal cancer (CRC) is a major contributor to cancer-related mortality in the United States and remains a substantial public health challenge worldwide. Although screening modalities have proven efficacy in reducing both incidence and mortality, adherence to these preventive measures is still suboptimal. We present a Hispanic man in his early 50s with no prior screening who developed CRC.

Our patient presented to the emergency department with a one-month history of chronic left upper quadrant abdominal pain, diarrhea, unintentional weight loss, decreased appetite, intermittent fevers, and an extensive history of cancer in his family. Investigations revealed a large, partially obstructive mass in the splenic flexure with extramural extension. He underwent an extended left colectomy with partial gastrectomy and loop ileostomy. Pathology confirmed low-grade colorectal adenocarcinoma. He reports no regular medical care and no prior esophagogastroduodenoscopy or colonoscopy.

This case demonstrates an atypical presentation with left upper quadrant pain relating to splenic flexure involvement and the absence of rectal bleeding. It highlights the need to maintain a high index of suspicion for colorectal malignancy in patients with persistent or unexplained GI symptoms, even when the clinical presentation is atypical or localizes outside the expected distribution.

## Introduction

Colorectal cancer (CRC) arises from a complex interaction of genetic susceptibility, environmental exposures, and lifestyle-related factors. It also represents a major contributor to cancer-associated morbidity and mortality worldwide [1]. Tumors involving the splenic flexure are rare and may present with less specific symptoms like obstipation, abdominal distention, and pain, as well as absence or minimal presence of bloody stools, also known as hematohezia [2]. This case highlights the atypical presentation of CRC in a middle-aged man at advanced stages, even in the absence of classic symptoms such as rectal bleeding, and underscores the critical importance of timely screening for early detection and improved outcomes.

## Case presentation

A Hispanic man in his early 50s with no significant past medical or surgical history presented to the emergency department with a long-standing history of intermittent stabbing left upper quadrant abdominal pain that has worsened over the last month, along with non-bloody diarrhea. He also reported significant unintentional weight loss and decreased appetite due to the worsening of his symptoms. He denied nausea, vomiting, melena, or hematochezia but reported intermittent fevers for the last month. He reported a history of prior heavy alcohol use of approximately 24 beers on the weekends but quit drinking six years earlier. The patient had no history of tobacco or illicit drug use. Family history was notable for leukemia in his mother, gastric and breast cancer in his maternal aunts, and osteosarcoma in his nephew. He reported that he had no regular medical care and never had an esophagogastroduodenoscopy or colonoscopy done. However, he was admitted to the hospital three months prior due to cellulitis of the lower extremity. On exam, he was alert and oriented, vital signs were within normal limits, and there was mild tenderness in the left upper quadrant. There was no jaundice, ascites, or peripheral edema.

Upon arrival at the emergency department, initial laboratory investigations are demonstrated in the table below (Table 1). These findings suggested some type of chronic inflammatory process, which raised concern for underlying malignancy, specifically in the context of the significantly elevated tumor marker levels.

**Table 1:** Lab values upon admission to hospital

Test	Patient result	Reference range
Hemoglobin	11.4 g/dL	13.5-17.5 g/dL
Hematocrit	38.70%	40-52%
Red blood cell count	5.66 x10 ^ 6	4.5-5.9 ×10⁶/μL
Mean corpuscular volume	68.4	80-100 fL
Mean corpuscular hemoglobin	20.1	27-33 pg
Mean corpuscular hemoglobin concentration	29.5	32-36 g/dL
Red cell distribution width	19.8	11.5-14.5%
Platelet count	488 ×10⁹/L	150-400 ×10⁹/L
Carcinoembryonic antigen	63.3 ng/mL	0-3.4 ng/mL
Serum creatinine	0.8	0.6-1.3 mg/dL
Blood urea nitrogen	11	7-20 mg/dL
Estimated glomerular filtration rate	>90	≥90 mL/min/1.73 m²
Sodium sodium	138	135-145 mmol/L
Potassium	5.3	3.5-5.0 mmol/L
Chloride	104	96-106 mmol/L
Bicarbonate	25	22-28 mmol/L
Aspartate aminotransferase	40	10-40 U/L
Alanine aminotransferase	31	7-56 U/L
Alkaline phosphatase	74	40-130 U/L
Total bilirubin	1	0.1-1.2 mg/dL
Albumin	4	3.5-5.0 g/dL
Total protein	7.9	6.0-8.3 g/dL

**Figure 1:**
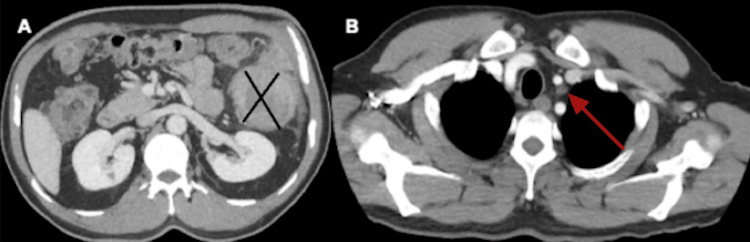
Cross-sectional imaging findings (A) Contrast-enhanced CT of the abdomen demonstrating a large, partially obstructive mass at the splenic flexure with extramural extension. (B) CT of the chest showing mild mediastinal lymphadenopathy, without radiographic evidence of distant metastatic disease. CT: computed tomography

A CT scan of the abdomen and pelvis revealed a 7.6 × 7.3 cm partially obstructive mass at the splenic flexure with extramural extension into the adjacent pericolonic and omental fat (Figure 1A)​​​. The staging workup with chest CT demonstrated mild mediastinal lymphadenopathy measuring 6.7 mm (Figure 1B).

**Figure 2:**
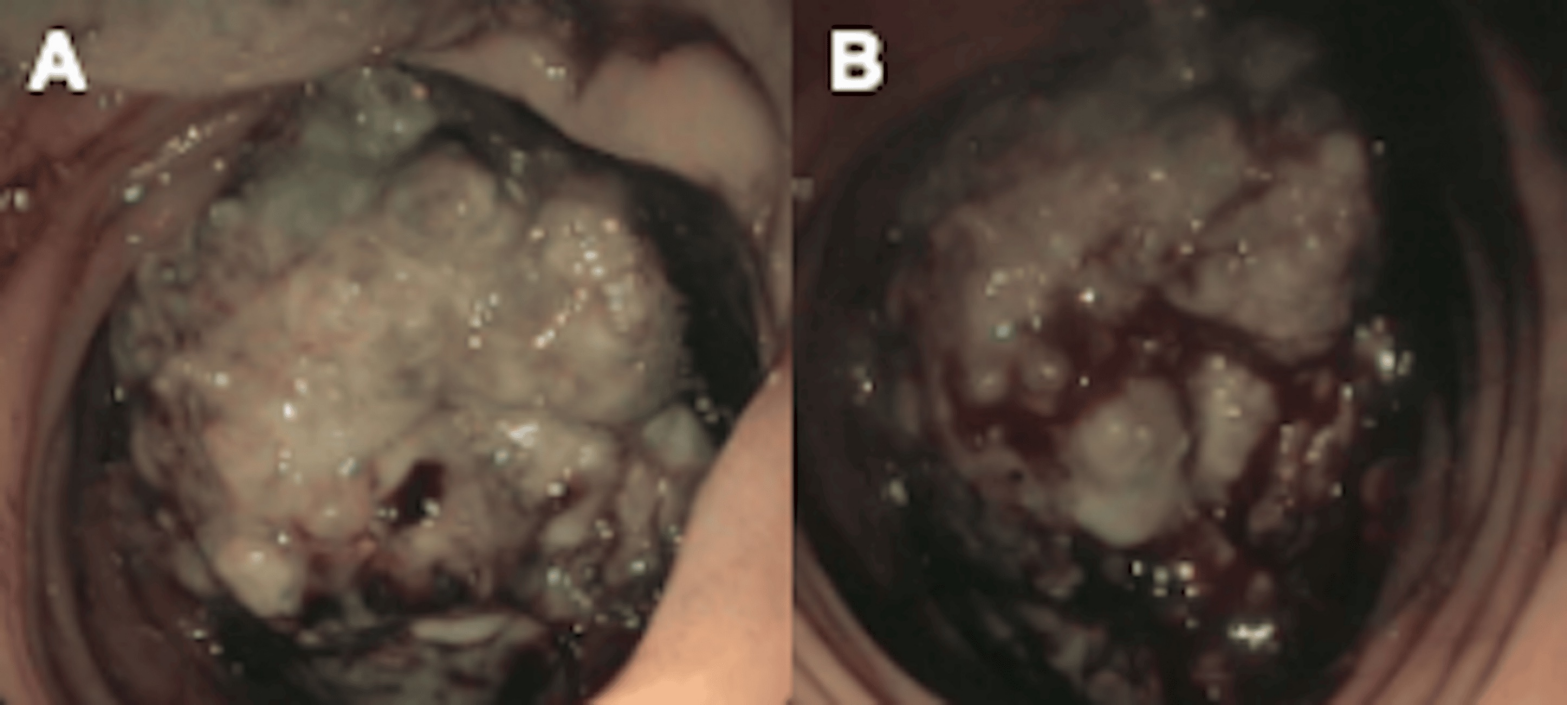
Colonoscopic appearance of a splenic flexure mass (A) Fungating, ulcerated mass causing partial obstruction of the colonic lumen. (B) Close-up view demonstrating friable tumor surface with necrosis and mucosal irregularity.

Colonoscopy further characterized the lesion as a fungating, infiltrative, and ulcerated mass measuring approximately 10 cm in length and causing partial luminal obstruction at the splenic flexure, which raised concern for malignancy rather than benign inflammation (Figure 2A-2B).

**Figure 3:**
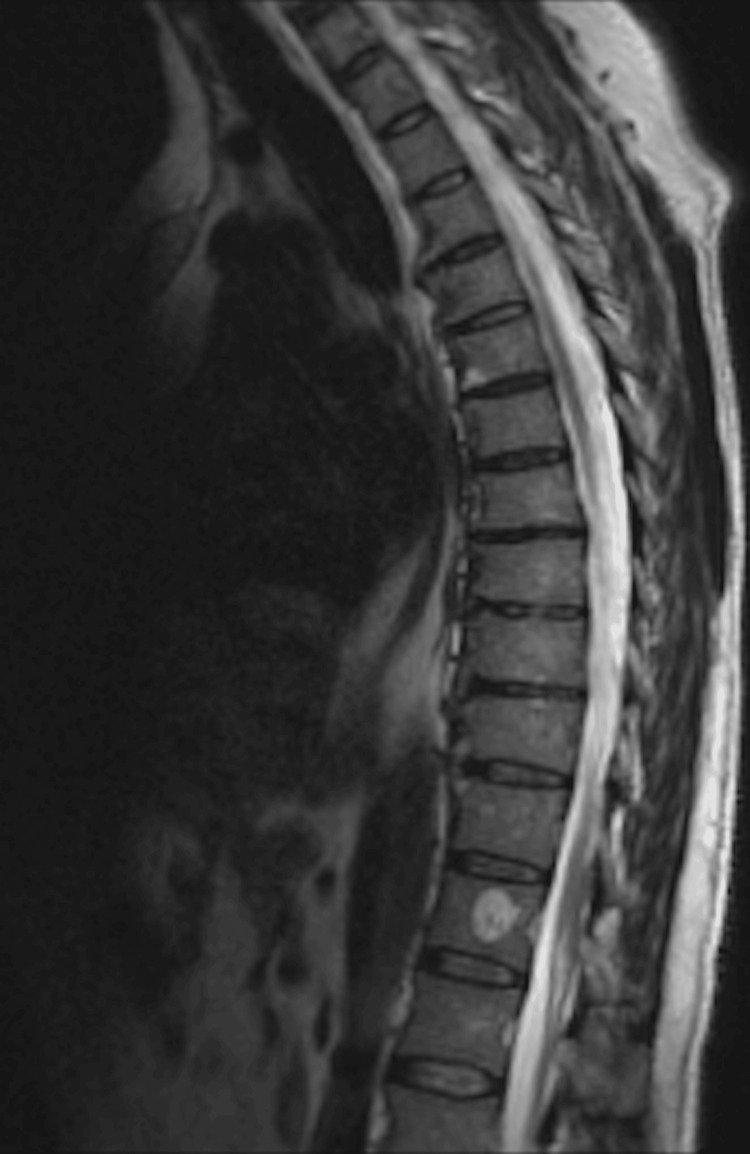
MRI of the thoracic and lumbar spine Sagittal MRI demonstrating degenerative changes without evidence of osseous or epidural metastatic disease.

MRI of the thoracic and lumbar spine showed mild degenerative disease in the lumbar spine, worse at L5-S1, with mild canal narrowing; however, it was negative for metastatic disease (Figure 3).

**Figure 4:**
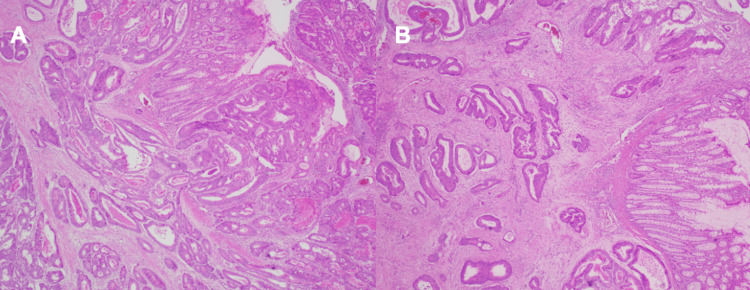
Histopathological features of colorectal adenocarcinoma from the patient's biopsy specimen (original image) (A) Low-power H&E staining demonstrating infiltrative, irregular glandular architecture consistent with adenocarcinoma. (B) Higher-power view showing well-to-moderately differentiated malignant glands embedded within a desmoplastic stromal background. H&E: hematoxylin and eosin

Integration of these findings, specifically elevated serum carcinoembryonic antigen (CEA), tumor size, and imaging of extramural involvement, indicated that the stage of disease was likely stage 2 or 3, thereby emphasizing the need for surgical resection and adjuvant therapy. The patient underwent open extended left colectomy with en bloc abdominal wall resection and partial gastrectomy, creation of side-to-side functional anastomosis, open splenic flexure mobilization, open creation of loop ileostomy, and open appendectomy. Histopathological examination of the surgical resection specimen confirmed a low-grade (well to moderately differentiated) colorectal adenocarcinoma (Figure 4A-4B). Postoperatively, he recovered without immediate complications and is planned for adjuvant therapy along with long-term surveillance via colonoscopy and imaging.

This case highlights several important points: First, the atypical presentation of CRC tumors, such as the left upper quadrant pain and non-bloody diarrhea without the classic symptom presentation of CRC, can contribute to delayed recognition. Second, laboratory abnormalities, such as microcytic anemia, thrombocytosis, and elevated CEA, provided early evidence suggestive of malignancy and inflammation, which ultimately influenced the staging and surgical planning. Finally, the correlation among imaging, endoscopic findings, and intraoperative assessment underscores the importance of a multimodal diagnostic approach for accurate staging and optimal surgical management, consistent with the current literature on atypical presentations and outcomes.

## Discussion

CRC remains a prevalent malignancy worldwide and continues to represent a substantial cause of cancer-related mortality, particularly in high-income countries [1,3]. In the United States, approximately 153,000 new cases of CRC and 52,550 deaths were estimated in 2023 [3]. More recent data from 2025 reports approximately 154,270 newly diagnosed cases, accounting for 7.5% of all new cancer cases in the country. During the same year, an estimated 52,900 deaths from CRC occurred, accounting for 8.6% of all cancer-related deaths [4]. These numbers demonstrate a persistent and gradual increase in both incidence and deaths despite the advances in screening and treatment, underscoring the ongoing impact of CRC on public health.

Guidance from U.S. professional organizations, including the United States Preventive Services Task Force (USPSTF) and the American Cancer Society, supports routine CRC screening beginning at age 45 for the average-risk population [1]. Currently, available screening modalities include colonoscopy every 10 years, annual fecal immunochemical testing (FIT), and FIT-DNA every one to three years. Screenings can potentially reduce CRC mortality by up to 60% through early detection [4]. Among available screening modalities, colonoscopy offers the advantage of direct visualization and concurrent removal of premalignant lesions [5]. However, the effectiveness of these interventions depends on patient participation and appropriate diagnostic follow-up after abnormal screening results, both of which remain inconsistent across clinical settings.

CRC is more frequently diagnosed in men than in women. It exhibits higher incidence rates in developed regions, with North America ranking among the areas with the most significant burden of both colon and rectal cancers [6]. However, Primm et al. have shown that the CRC burden is not evenly distributed across racial/ethnic groups, particularly in incidence and stage at diagnosis. Studies found that Black, American Indian, and Hispanic patients were more likely to be diagnosed at further progressed stages of disease, despite advances in screening techniques and treatment [7]. Stage-specific analyses demonstrated that poorer cancer-specific survival among Black, Hispanic, and Pacific Islander patients persisted even in early-stage disease, when therapeutic interventions are typically most effective, suggesting inequities throughout the cancer care continuum, including screening, treatment, and post-treatment surveillance [7].

The present case illustrates an atypical manifestation of CRC with left upper quadrant pain and non-bloody diarrhea, emphasizing the importance of considering malignancy even when typical features are absent. Splenic flexure tumors (SFTs) are relatively uncommon, accounting for approximately 2-5% of colorectal tumors [8], leaving their characteristics poorly defined. SFTs are typically defined as being located between the distal third of the transverse colon and the proximal segment of the descending colon [9]. These types of CRC are more likely to present with vague or atypical symptoms compared with right- or sigmoid-sided lesions. The most common clinical manifestation is reported as abdominal pain, one of the main symptoms that our patient presented with [10]. Other possible symptoms that were reported include constipation, diarrhea, obstipation, rarely the presence of bloody stools or rectal bleeding, and no weight loss [4]. SFTs are also found to occur more frequently in males, are diagnosed at a younger age, and are more likely to present with obstruction and be larger in size compared to other types of colorectal tumors [10].

Our patient was also notable for intermittent fevers without the classic GI symptoms typically associated with CRC. While most patients with colorectal malignancies present with changes in bowel habits, rectal bleeding, abdominal pain, or weight loss, fevers are uncommon and can delay diagnosis. CRC has rarely been reported in the literature as an underlying cause of fever of unknown origin. Dai and Chung reported cases of colon carcinoma presenting with prolonged, recurrent fevers (38.3-38.8°C) without specific GI symptoms, supporting fever as a rare paraneoplastic manifestation of CRC [11]. Similarly, our patient lacked classic alarming features and presented only with nonspecific left upper quadrant pain, non-bloody diarrhea, and intermittent fevers. Furthermore, underscoring the atypical presentation and diagnostic challenges of SFTs.

Disease stage, tumor characteristics, and patient-specific factors guide management strategies for CRC. Table 2 summarizes stage-based treatment approaches and post-treatment surveillance recommendations for classic presentations of CRC, with local excision or surgical resection as the most common primary management [12]. In SFTs, challenges arise especially in surgical management due to their anatomical position, vascular supply, and lymphatic drainage, which sit at the colon's watershed area between the superior and inferior mesenteric arterial areas [9,13].

**Table 2:** Stage-based management and surveillance recommendations for CRC Adapted from NCCN Guidelines for Patients: Colon Cancer [12]. CEA: carcinoembryonic antigen, CT: computed tomography, T4: tumor stage 4, NCCN: National Comprehensive Cancer Network, CRC: colorectal cancer

Stage	Primary treatment approach	Post-treatment surveillance
Stage 0 (carcinoma in situ)	Local excision or polypectomy with curative intent when complete resection is achieved.	Colonoscopic surveillance according to polyp follow-up guidelines. Repeat colonoscopy at 1 year if high-risk features or incomplete examination are present; otherwise, repeat at 3 years and then every 5 years if normal. No routine CEA testing or imaging is recommended.
Stage I	Surgical resection is typically a segmental colectomy with regional lymph node evaluation. Adjuvant therapy is not routinely indicated.	Colonoscopy at 1 year following surgery. If normal, repeat at 3 years, then every 5 years. Routine imaging or CEA monitoring is not recommended in the absence of symptoms.
Stage II	Surgical resection is the primary treatment. Consideration of adjuvant chemotherapy is based on high-risk pathologic features such as T4 disease, perforation, obstruction, or poor differentiation.	Clinical evaluation and CEA testing every 3–6 months for the first 2 years, then every 6 months up to 5 years. CT imaging of the chest, abdomen, and pelvis every 6–12 months for up to 5 years. Colonoscopy at 1 year postoperatively, then at 3 years and every 5 years thereafter if normal.
Stage III	Surgical resection followed by adjuvant chemotherapy, typically administered for 3–6 months, depending on risk stratification.	Surveillance mirrors Stage II recommendations, including regular clinical assessment, CEA monitoring, interval CT imaging, and colonoscopic follow-up.
Stage IV (metastatic)	Resectable disease may be managed with colectomy and metastasectomy with or without perioperative chemotherapy. Unresectable disease is treated with systemic chemotherapy, with or without targeted or biologic agents, and palliative interventions as appropriate.	Following curative-intent treatment, surveillance is similar to Stage II–III disease, with close clinical follow-up, serial CEA testing, interval cross-sectional imaging, and scheduled colonoscopy. More intensive surveillance is recommended due to a higher risk of recurrence.

In the present case, the patient’s tumor demonstrated extramural extension at the splenic flexure with partial luminal obstruction and a size of approximately 10 cm, corresponding to Stage II disease with high-risk features. Consistent with the recommendations outlined in Table 2, he underwent surgical resection with an open extended left colectomy and en bloc resection of adjacent structures, followed by plans for adjuvant therapy and surveillance. By explicitly linking clinical findings to guideline-directed treatment, this case exemplifies the practical application of stage-based management in complex SFTs. It underscores the importance of individualized surgical planning for tumors with anatomically challenging features.

Given the substantial morbidity and mortality associated with CRC, early recognition of both classic and atypical clinical presentations, including uncommon sites such as SFTs, is essential for timely diagnosis and intervention. Our case, presenting with left upper quadrant pain, non-bloody diarrhea, and intermittent fevers without classic “red flag” symptoms, exemplifies the atypical presentation that is uniquely reported in SFTs. Continued efforts to promote public awareness of preventive strategies and treatment remain vital to reducing the overall burden of CRC.

## Conclusions

CRC remains a leading cause of morbidity and mortality, particularly in the context of modifiable lifestyle and inherited risk factors. While classic presentations often include rectal bleeding and changes in bowel habits, this case, characterized by left upper quadrant pain, non-bloody diarrhea, and intermittent fevers, illustrates how CRC can present atypically and in rare locations of the colon. Delayed or absent screening likely contributed to disease progression, underscoring the critical importance of adherence to USPSTF screening guidelines. Early detection through colonoscopy and polyp removal remains central to prevention and improved outcomes. This case reinforces the need to consider CRC in atypical GI presentations and reinforces the pivotal role of screening in reducing disease burden and mortality.
